# Global Mapping of Research Trends on Interventions to Improve Health-Related Quality of Life in Asthma Patients

**DOI:** 10.3390/ijerph17103540

**Published:** 2020-05-19

**Authors:** Hai Thanh Phan, Giap Van Vu, Giang Thu Vu, Giang Hai Ha, Hai Quang Pham, Carl A. Latkin, Bach Xuan Tran, Cyrus S.H. Ho, Roger C.M. Ho

**Affiliations:** 1 Institute for Preventive Medicine and Public Health, Hanoi Medical University, Hanoi 100000, Vietnam; 020101190574@daihocyhanoi.edu.vn (H.T.P.); bach.ipmph@gmail.com (B.X.T.); 2 Department of Internal Medicine, Hanoi Medical University, Hanoi 100000, Vietnam; vuvangiap@hmu.edu.vn; 3 Respiratory Center, Bach Mai Hospital, Hanoi 100000, Vietnam; 4 Center of Excellence in Evidence-based Medicine, Nguyen Tat Thanh University, Ho Chi Minh City 700000, Vietnam; giang.coentt@gmail.com; 5 Institute for Global Health Innovations, Duy Tan University, Da Nang 550000, Vietnam; phamquanghai@duytan.edu.vn; 6 Faculty of Pharmacy, Duy Tan University, Da Nang 550000, Vietnam; 7 Faculty of Medicine, Duy Tan University, Da Nang 550000, Vietnam; 8 Bloomberg School of Public Health, Johns Hopkins University, Baltimore, MD 21205, USA; carl.latkin@jhu.edu; 9 Department of Psychological Medicine, National University Hospital, Singapore 119074, Singapore; cyrushosh@gmail.com; 10 Institute for Health Innovation and Technology (iHealthtech), National University of Singapore, Singapore 119077, Singapore; pcmrhcm@nus.edu.sg; 11 Department of Psychological Medicine, Yong Loo Lin School of Medicine, National University of Singapore, Singapore 119228, Singapore; 12 Center of Excellence in Behavioral Medicine, Nguyen Tat Thanh University, Ho Chi Minh City 700000, Vietnam

**Keywords:** scientometrics, content analysis, text mining, interventions, asthma, quality of life, HRQoL

## Abstract

Globally, approximately 335 million people are being affected by asthma. Given that asthma is a chronic airway condition that cannot be cured, the disease negatively impacts physical health and results in losses of productivity of people experiencing asthma, leading to decrease in quality of life. This study aims at demonstrating the research trends worldwide and identifying the research gaps in interventions for improving quality of life of patients with asthma. Bibliometric approach and content analysis, which can objectively evaluate the productivity and research landscapes in this field, were utilized. In this study, we systematically quantified the development of research landscapes associated with interventions for improving quality of life of people experiencing asthma. Along with the gradual growth in the number of publications, these research topics have relatively expanded in recent years. While the understanding of the pathophysiology, diagnosis and treatment of asthma has been well-established, recent research has showed high interest in the control and management of asthma. Findings of this study suggest the need for more empirical studies in developing countries and further investigation into the effects of environment factors on asthma outcomes, as well as the economic burden of asthma.

## 1. Introduction

According to the Global Initiative for Asthma (GINA), asthma is a heterogeneous disease characterized by chronic airway inflammation [1]. The hallmark features of asthma include reversible airflow obstruction, airway eosinophilia, and history of recurrent wheeze along with cough and breathlessness [1,2]. The pathophysiology of asthma involves various cells and cellular elements, such as mast cells, eosinophils, T lymphocytes, macrophages, neutrophils, and epithelial cells. The inflammation not only causes recurrent episodes of cough, wheezing, shortness of breath, and chest tightness, but also results in an associated increase in the existing bronchial hyperresponsiveness to various stimuli [3]. 

Globally, approximately 335 million people are being affected by asthma, and in 2015 only, about 383,000 deaths were attributed to asthma [4,5]. Asthma is ranked 14th among the most serious disorders due to its negative impacts on the people experiencing the disease, and its economic burden on healthcare facilities and governments [6]. Asthma has been observed in both children and adults. The incidence and prevalence of pediatric asthma appear to be higher, while morbidity and mortality are more common among adults. Children with asthma may have impaired airway development as well as reduced maximally attained lung function, which may persist into adulthood without further progressive loss. Meanwhile, in adults, asthma usually facilitates a decline in lung function and enhances the risk of fixed airflow obstructions, especially for asthmatics who smoke [7]. Asthma is also associated with a number of respiratory comorbidities, namely rhinosinusitis, allergen rhinitis, sleep-disordered breathing in children, and chronic obstructive pulmonary disease (COPD) and chronic sinusitis for adults. Such comorbidities may not only somewhat enhance the asthma symptoms but also complicate clinical care in various ways [8,9,10]. 

Since traditional measures of asthma outcome, such as pulmonary functions and respiratory symptoms, are insufficient to demonstrate the limitations that asthma causes to patients, subjective experience of health-related quality of life of patients plays a critical role in the evaluation of interventions’ effectiveness [11,12,13]. A number of factors have been reported to have an association with poor quality of life among people suffering from asthma, including sociodemographic characteristics (higher age, female gender, lower education level and unemployment), clinical conditions (severity, hospitalization, high levels of immune markers), poor control and management, and associated comorbidities [14,15]. Therefore, conceptualized healthcare beyond medical treatment is crucial for individuals living with asthma, who need to be able to deal with and manage the symptoms themselves. The evaluation of health-related quality of life among patients with asthma is beyond a mere measurement of their situation and healthcare needs, as it also makes a great contribution to the assessment of the effectiveness of clinical interventions.

In addition to pharmacological treatment, including the use of bronchodilator and inhaled corticosteroids (ICS), or biological therapy, such as omalizumab, mepolizumab, and reslizumab, interventions to improve quality of life of asthmatics involve a personalized and comprehensive approach [1,2,3]. Self-management, namely training in proper use of inhaler for children as well as caregivers, family members, and teachers, and writing asthma action plans are among common interventions [1]. Since incorrect practice of using inhaler is highly common and usually results in an increased risk of asthma attacks, inhaler training with physical demonstration does play a crucial role. Regularly repeated inhaler training has been proved to improve the level of asthma control in adult patients [16]. Along with self-monitoring, the importance of a written asthma action plan that guides the patients and their caregivers on how to promptly recognize and make correct responses to asthma exacerbations is undeniable. Such education has helped to reduce up to two-thirds of urgent healthcare, work and school absenteeism, and even night waking [17,18]. Other approaches target comorbidities and/or modifiable risk factors, such as reducing the use of aspirin or other non-steroidal anti-inflammatory drugs (NSAIDs) in patients with aspirin-exacerbated respiratory disease, and avoidance of exposure to tobacco smokes, occupational pollution, and mold or damp [1]. 

This study aims at demonstrating the research trends worldwide and identifying the research gaps in interventions for improving quality of life of patients with asthma. In order to report the trend in available articles over time and measure the global research growth based on the existing literature, we applied a bibliometric approach and content analysis, which can objectively evaluate the productivity and research landscapes generated by researchers, health professionals and institutions in this field. By pointing out the current research patterns, we are able to examine the development as well as productivity, and identify research gaps of the literature on quality of life among people suffering from asthma, and thus better inform health professionals worldwide.

## 2. Materials and Methods 

### 2.1. Search Strategy

The data were retrieved from the Web of Science (WoS) Core Collection, which covers a large number of scientific domains and technology fields. WoS provides publications from high-quality scientific journals, which have been accessed by the experts of literature review committees. WoS is also among the databases with widest coverage, with citation and bibliographic data going back until the 1900s [19]. 

The search strategy can be described as follows:

- Step 1: With the use of Boolean operators “OR”, the search query was developed to identify the number of published items included “quality of life” OR “QoL” OR “HRQoL” OR “well-being”. We downloaded papers in text format (txt.) and imported to STATA for further extraction.
- Step 2: Among papers found in Step 1, we used STATA syntax to filter the papers with the following terms in titles or abstracts: (“asthma” OR “asthmatic*”) AND (“intervention*” OR “trial*”). Papers that did not mention “asthma” in their titles and abstracts were removed at this step.
- Step 3: We screened the abstracts of the papers from Step 2 to figure out the papers with asthmatics as study participants and which mentioned quality of life as an outcome. The process was performed by two independent researchers. Any arisen disagreement was solved by discussing with the research team and senior researchers.

### 2.2. Data Download and Extraction

In addition to titles and abstracts, metadata of each paper and reports generated by WoS database were downloaded in text format and imported in Excel for analyzing, including:

1. Total number of publications
2. Authors’ names, their affiliations and the number of total papers, and total citations for each author
3. Most prolific countries and collaborations
4. Institutional affiliations and frequency of citation
5. The top cited articles with titles, authors, journal details, year of publication, total citations and citation per year.
6. Titles, abstracts and keywords

Selected papers were research articles written in English. Grey literature, conference proceedings, reviews and books/book chapters were excluded. As the search was performed in the middle of 2019, only papers published before and in the year 2018 were included in the analysis. 

### 2.3. Data Analysis

We used STATA version 15.0 (STATACorp., College Station, TX, USA) to analyze the final data in step 4 using the following information on the articles: authors’ affiliations, titles, journals’ names, keywords, total number of citations, and abstracts.

Publications’ general characteristics, namely year of publication, the number of papers per year of each country, accumulative citations from published year to 2018, yearly mean citation rate, total usage in the last six months and the last five years, and mean use rate in the last six months and the last five years, were described. We used STATA to calculate the number of papers by country in abstracts. 

A network graph showing the co-occurrence of terms in title and abstracts was established by VOSviewer (version 1.6.11, Center for Science and Technology, Leiden University, Leiden, The Netherlands). A co-occurrence network can be described as the collective interconnection of terms and is generated by connecting pairs of terms based on a set of criteria that defines co-occurrence. In this study, when term A and term B both appear in title and/or abstract of a particular publication, they are said to “co-occur”. Additionally, another paper may contain terms B and C, and so on. By linking the co-occurrence of the identified terms, we created a co-occurrence network of terms.

Latent Dirichlet Allocation (LDA) was applied to classify papers into corresponding topics. LDA is a common topic modeling algorithm for text mining to determine the relationships of text documents. In LDA, each term is regarded as a random vector with the probability of drawing the words/texts associated with that term, in other words, that vector. The probability of a paper is calculated based on the probability of the terms, and papers with similar probability will be classified into one group [20,21,22,23,24,25]. Thus, by applying LDA, we would be able to recognize the interdisciplinary structure of research development as well as obtain an in-depth view of hidden themes of research on interventions in asthma [26]. We reviewed and read through the most cited papers of each group and assign the labels for each topic manually. In addition, to analyze the relationship of the research area, we utilized coincidence analysis using the STATA command ‘precoin’ and presented the results in the form of a dendrogram. Dendrograms graphically illustrate the information concerning which observations are grouped together at various levels of similarity.

Analytical techniques for each data types are summarized below (Table 1):

## 3. Results

### 3.1. Number of Published Items and Publication Trend 

**Figure 1:**
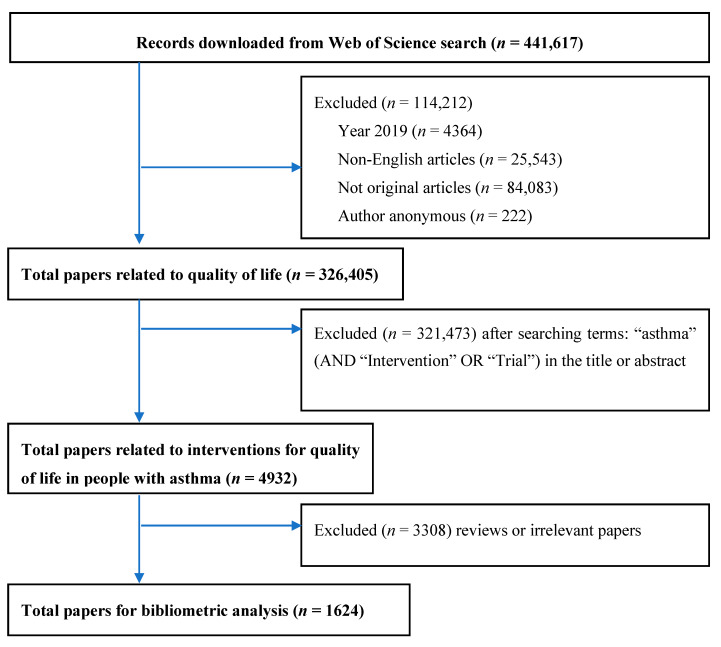
Selection of papers.

The process of screening and selecting papers is presented in Figure 1. Among 326,405 articles about quality of life, a total of 1624 publications met the eligible criteria and was selected for further analysis. 

General characteristics of the selected publications are presented in Table 2. Since 1991, when the first studies on quality of life of people suffering from asthma were published, the number of papers considering this research interest has grown gradually. The total citations of articles published during the 2000s were relatively high compared to publications in recent years. Meanwhile, the total usage, defined by the total downloads, and mean use rate during the last six months were considerable for the research in 2017 and 2018, suggesting the continuous update and rising interest in the topic recently.

### 3.2. Number of Study Settings by Countries 

Table 3 show the number of papers categorized by the countries of the study settings as mentioned in abstracts. There was a total of 407 articles describing locations of settings, and 14.3% of these were set up in the United States, making this country the top of the list. Over half of the study settings (55.6%) belonged to the top 10 countries, which are all classified as developed countries. 

### 3.3. Thematic Analysis of Literature and Research Interests Over Time

**Figure 2:**
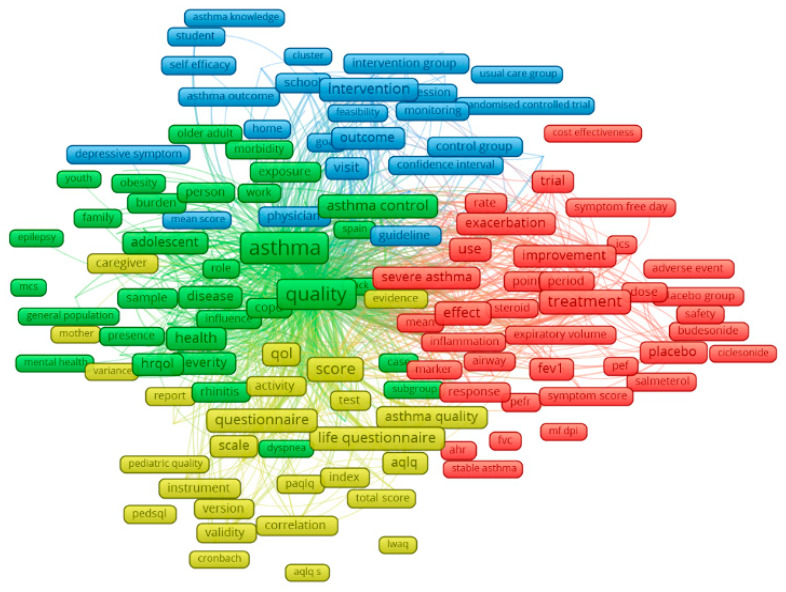
Co-occurrence of most frequent terms in titles and abstracts. Note: the colors of the nodes indicate principle components of the data structure; the node size was scaled to the keywords’ occurrences. Abbreviations: (red cluster) fvc—forced vital capacity, fev1—forced expiratory volume in one second, pef—peak expiratory flow, pefr—peak expiratory flow rate, ahr—airway hyperresponsiveness, ics—inhaled corticosteroids, mf dpi—mometasone furoate dry powder inhaler; (yellow cluster) qol—quality of life, aqlq—Asthma Quality of Life Questionnaire, aqlq s—Asthma Quality of Life Questionnaire (Standardized), paqlq—Pediatric Asthma Quality of Life Questionnaire, pedsql—Pediatric Quality of Life Inventory, lwaq—Living with Asthma Questionnaire; (green cluster) mcs—multiple chemical sensitivity.

Figure 2, which was generated by the analysis of content of abstracts and titles, provides an illustration of the most frequently co-occurred groups of terms. There were four clusters emerging from 458 terms with co-occurrence at least 30 times. Red cluster points out indicators of pulmonary function tests, such as forced vital capacity (FVC), forced expiratory volume in one second (FEV1), peak expiratory flow (PEF), and treatment for different asthma conditions, including inhaled corticosteroids (ICS), mometasone furoate dry powder inhaler (MF-DPI), salmeterol, etc. Yellow cluster focuses on common questionnaires for measuring asthma-related quality of life, namely Asthma Quality of Life Questionnaire (AQLQ), Pediatric Quality of Life Inventory (PedsQL) and Living with Asthma Questionnaire (LWAQ). Blue nodes reveal various approaches of intervention for asthma, while green cluster refers to comorbidity of asthma and factors associated with quality of life among asthmatics. 

**Figure 3:**
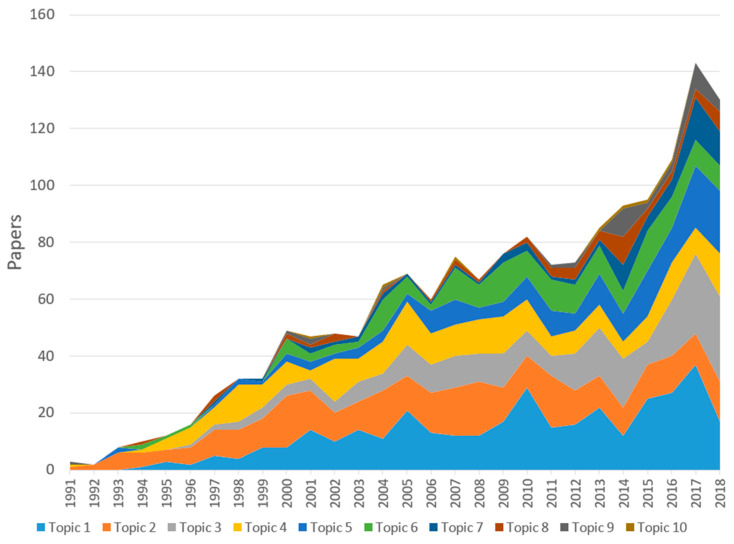
Changes in research topics development.

By applying Latent Dirichlet Allocation for the abstracts, we constructed research articles into ten major topics (Table 4, Figure 3). We extracted a list of papers most likely to be associated with each topic identified, then reviewed the titles and abstracts of the most cited papers within each group to determine the name for each topic. The topic number was assigned by the number of papers belonging to the topic in the last five years. The *n* (%) is the total number and percentage of papers classified into the topic over the research period. We also rank the topics by highest literature volume produced in the last five years. According to the results of the LDA analysis, asthma control and self-management education interventions was the topic of most interest over time as well as in the last five years, accounting for over one-fifth of the literature volume. Meanwhile, the total number of studies on application and impact of non-medical treatment og asthma have received relatively little attention. 

In addition, Figure 3 provides an evident illustration of the trend in the interest in each topic throughout the research period. Despite being ignored in the first years, research on asthma control and education interventions for asthmatics (Topic 1) has increased rapidly and become the research topic that covers a large body of the literature, with a robust rise, especially in recent years. Studies into specific types of asthma (Topic 2 and Topic 7), and the association between diet and exercise and quality of life of people with asthma (Topic 3), albeit having a modest start, have been significantly increasing in recent years. By contrast, as the instruments for measuring asthma-related quality of life, to a certain extent, have become well-established and proved their validity, there has been a slight decline of research volume for Topic 4.

**Figure 4:**
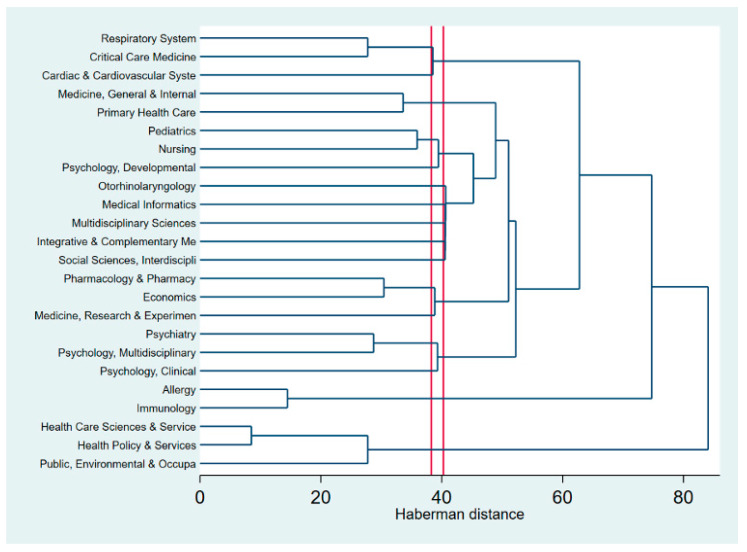
Dendrogram of coincidence of research areas using the Web of Science (WoS) lassifications.

Figure 4 presents the dendrogram, in other words, the hierarchical clustering of research disciplines in quality of life of patients with asthma. The distance or dissimilarity between clusters is indicated in the horizontal axis, while the vertical axis shows the research disciplines. The red lines show the depth for the cut-off of the analysis [27]. According to Figure 4, the research landscapes in quality of life among patients with asthma are rooted in the following disciplines: (a) Health policy and services, (b) Health care sciences and services and (c) Public, Environmental and Occupational health, as the first chunk in the bottom the dendrogram. In the top part, we found the integration of: (a) Respiratory system, (b) Critical care medicine, and (c) Cardiac and Cardiovascular system. 

## 4. Discussion

### 4.1. Summary of Findings

This study provides a quantitative as well as qualitative overview of interdisciplinary research landscapes of quality of life among individuals with asthma. In this study, we have systematically quantified the development of research landscapes associated with interventions for improving quality of life of asthmatics in the past 28 years. While the number of publications showed an insignificant change over years, the substantial total usage and mean use rate in the last six months of studies published in 2017 and 2018 implies an increasing interest and the need for a continuous update of understandings of this topic. Along with the growth in the number of publications, the research topics have also been expanded in recent years. Besides the conventional research areas such as development of instruments and treatment, asthma control and education interventions for people living with asthma, research into specific types of asthma have also emerged and attracted a great deal of attention. 

### 4.2. Current Research on Asthma in Different Countries 

In terms of the countries involved in the research area, the top nine countries with the highest number of study settings belonged to the high-income group [28]. In addition to the United States, which led in research on interventions to improve quality of life of asthmatics, a large number of study settings on quality of life of people living with asthma took place in Australia, the United Kingdom and Europe (Table 2). These efforts have resulted in the stabilized or decreasing prevalence of asthma in such developed countries, as opposed to developing countries, which have been facing a steep increase in the burden of asthma [4,29,30]. It is also noteworthy that low- and lower-middle income countries accounted for more than 80% of asthma-related deaths worldwide, which calls for more empirical studies to be conducted in developing countries [5]. 

### 4.3. Emerging Research Interests

Realizing the growing importance of asthma control in improving quality of life of people with asthma at a population level, one of the emerging research domains that has attracted much attention from researchers in recent years is asthma control and education interventions for asthmatics (Topic 1—Table 3, Figure 3). Aside from clinical history and manifestations, namely experience of near-fatal asthma, sensitivity to mold and other allergens, and comorbidities with other respiratory diseases and infections, poor management and control of asthma has been reported to be responsible for a large number of asthma exacerbation and asthma-related preventable deaths [4,31,32,33,34,35,36,37,38,39]. Whilst understanding of the pathophysiology, diagnosis and treatment of asthma has been well-established (Figure 2), research on controlling the distribution of asthmatics and how to increase their access to appropriate and high-quality interventions for better quality of life remains relatively scarce. Despite the development of various medications and interventions to improve the quality of life of asthmatics, asthma management and control, especially in developing countries, has been facing such barriers as unaffordability of inhaled corticosteroids, inadequate education on asthma for the general population and poor infrastructure, which frequently leads to lack of adherence to treatment as well as lack of appropriate and prompt actions in case of asthma attack [4,38,40,41]. Self-management, including the use of healthy diet and physical exercises (Topic 3), is also an important component of asthma management [42,43,44,45]. Although studies have demonstrated the association between food allergy and pathogenesis of asthma, and dietary factors are reported to directly influence asthma outcomes regardless of level of allergy, a number of people suffering from asthma and their caregivers have not yet been made aware of the importance of a healthy and appropriate diet [31,44,46,47,48]. Meanwhile, exercise showed positive effects on asthma control and can be recommended for children and young adults [43,49]. Given that a person best understands his or her allergy and physical status, self-management, considering diet and exercise, therefore plays an important role in alleviating asthma symptoms and improving quality of life. 

### 4.4. Identified Research Gaps 

Based on the results obtained, we have identified certain research gaps. Even though understanding of the pathogenesis of asthma has been extensively studied, the association between asthma and environmental factors deserves more attention from researchers. Asthma has been long recognized as a chronic condition and with the proper treatment and management, the symptoms can be well controlled and asthma patients are able to live a full and rewarding life [5]. Nevertheless, under the influence of continuously worsening air quality, it is undeniable that asthmatics have become more and more vulnerable [50,51,52,53]. Increased levels of ambient ozone, nitrogen dioxide (NO_2_), sulfur dioxide (SO_2_) and particulates (PM_2.5_, PM_10_) were proved to be associated with higher prevalence of asthma and symptoms onset, as well as increased hospital admission due to asthma attack [54]. Children and adolescents with asthma are particular susceptible to these pollutants due to their underdeveloped lung function and immature metabolic pathways [51,55,56,57]. The current environmental situation has showed little sign of improvement and has continued to bring about detrimental effects on health-related quality of life of asthma patients, suggesting the need for further update research on this topic. 

In the meanwhile, notwithstanding the fact that asthma is a chronic condition with both direct and indirect cost, the financial burden of asthma is not among the emerging research domains identified in this study. According to the Global Asthma Report 2018, annual direct cost per patient ranged from under USD 150 in United Arab Emirates to more than USD 3000 in USA and, at national level, total annual costs for asthma in the USA witnessed an increase of 3 billion USD from 2002 to 2011. Total costs for asthma patients aged 15 to 64 in Europe were USD 24.7 billion during 1999–2002, in which the United Kingdom itself accounted for USD 9.8 billion [4]. The data indicates that asthma is an economic burden even for high-income countries. While indirect costs are regularly ignored by cost estimates, studies have consistently indicated that indirect costs constitute a significant part of the economic burden of asthma [58,59,60]. On the other hand, high cost of treatment can be regarded as an important limiting factor for improving quality of life of asthma patients in developing countries. While access to inhaled corticosteroids has been recognized as one of the keys to improve quality of life among asthmatics, for many asthma patients in developing countries, such as India, Malaysia and Thailand, inhaled corticosteroids are either inaccessible or unaffordable [4,61,62]. In Indonesia, although inhaled corticosteroid is available and covered by health insurance, many people are unable to afford a spacer, which is the best delivery system for the drug [4]. Therefore, there presents a call for more research on the economic aspects of asthma. 

### 4.5. Limitations

In this study, we introduced an efficient approach to analyzing the extant literature about quality of life among asthmatics. Nonetheless, there exist some limitations. Even though a fairly large proportion of publications relating to quality of life is available on WoS, the choice of WoS as the only data source would leave out relevant studies. Another limitation that should be acknowledged is that the language of selected papers was restricted to English, potentially causing a bias toward English-speaking countries. Besides, instead of analyzing full texts, the content analysis and text mining involved solely titles and abstracts. Nevertheless, provided that the major content of a study is normally included in the title and abstract, this bibliometric analysis is able to provide meaningful findings and offer a comprehensive overview of current research trends.

## 5. Conclusions

In conclusion, by conducting a bibliometric analysis and applying a scientometric approach, we have presented the global research patterns and current interests and identified the research gaps in the literature relevant to quality of life among people suffering from asthma. Recent research has showed high interest in the control and management of asthma. Findings of this study suggest the need for more empirical studies in developing countries and further investigation into the effects of environment factors on asthma outcomes as well as the economic burden of asthma. 

## Figures and Tables

**Table 1:** Summary of techniques used for each type of data.

Type of Data	Unit of Analysis	Analytical Methods	Presentations of Results
Keywords, Countries	Words	Frequency of co-occurrence	Map of keywords clusters
Abstracts	Papers	Latent Dirichlet Allocation	10 classifications of research topics
WoS classification of research areas	WoS research areas	Frequency of co-occurrence	Dendrogram of research disciplines (WoS classification)

**Table 2:** General characteristics of publications.

Year Published	Total Number of Papers	Total Citations	Mean Cite Rate Per Year ^1^	Total Usage ^2^ Last 6 Months	Total Usage ^2^ Last 5 Years	Mean Use rate Last 6 Months ^3^	Mean Use Rate Last 5 Years ^4^
2018	129	229	1.78	228.00	364.00	1.77	0.56
2017	143	640	2.24	171.00	725.00	1.20	1.01
2016	109	888	2.72	70.00	718.00	0.64	1.32
2015	95	1056	2.78	49.00	677.00	0.52	1.43
2014	93	1185	2.55	37.00	785.00	0.40	1.69
2013	85	1710	3.35	43.00	996.00	0.51	2.34
2012	73	1467	2.87	24.00	664.00	0.33	1.82
2011	72	1880	3.26	23.00	475.00	0.32	1.32
2010	82	2259	3.06	44.00	509.00	0.54	1.24
2009	76	3302	4.34	33.00	464.00	0.43	1.22
2008	67	1833	2.49	11.00	280.00	0.16	0.84
2007	74	3357	3.78	29.00	359.00	0.39	0.97
2006	60	2601	3.33	22.00	271.00	0.37	0.90
2005	69	3399	3.52	18.00	274.00	0.26	0.79
2004	65	2539	2.60	13.00	203.00	0.20	0.62
2003	47	2009	2.67	12.00	161.00	0.26	0.69
2002	48	2528	3.10	11.00	191.00	0.23	0.80
2001	47	2559	3.02	6.00	160.00	0.13	0.68
2000	49	2366	2.54	8.00	187.00	0.16	0.76
1999	32	3088	4.83	9.00	96.00	0.28	0.60
1998	32	1764	2.63	1.00	53.00	0.03	0.33
1997	26	2001	3.50	4.00	84.00	0.15	0.65
1996	16	2268	6.16	5.00	57.00	0.31	0.71
1995	12	865	3.00	1.00	41.00	0.08	0.68
1994	10	1908	7.63	2.00	36.00	0.20	0.72
1993	8	1132	5.44	4.00	27.00	0.50	0.68
1992	2	1094	20.26	0.00	22.00	0.00	2.20
1991	3	469	5.58	0.00	7.00	0.00	0.47

^1^ mean cite rate per year = total citations/[total citations × (2018 − that year)]; ^2^ total usage: total downloads; ^3^ mean use rate last 6 months = total usage last 6 months/total number of papers; ^4^ mean use rate last 5 years = total usage last 5 years/(total number of papers × 5).

**Table 3:** Number of papers by countries as study settings.

	Country Settings	Frequency	%		Country Settings	Frequency	%
1	United States	58	14.3%	36	Cuba	2	0.5%
2	Australia	37	9.1%	37	Finland	2	0.5%
3	United Kingdom	32	7.9%	38	Georgia	2	0.5%
4	Sweden	18	4.4%	39	Greece	2	0.5%
5	Spain	17	4.2%	40	Hungary	2	0.5%
6	Netherlands	15	3.7%	41	Kuwait	2	0.5%
7	Canada	13	3.2%	42	Norway	2	0.5%
8	Japan	13	3.2%	43	South Africa	2	0.5%
9	Taiwan	12	2.9%	44	Sri Lanka	2	0.5%
10	Brazil	11	2.7%	45	Tunisia	2	0.5%
11	India	11	2.7%	46	United Arab Emirates	2	0.5%
12	Italy	11	2.7%	47	Wallis and Futuna	2	0.5%
13	China	10	2.5%	48	Argentina	1	0.2%
14	Germany	10	2.5%	49	Barbados	1	0.2%
15	France	9	2.2%	50	Benin	1	0.2%
16	Ireland	9	2.2%	51	Chile	1	0.2%
17	Saudi Arabia	7	1.7%	52	Czech	1	0.2%
18	Switzerland	7	1.7%	53	Dominica	1	0.2%
19	Egypt	6	1.5%	54	Dominican Republic	1	0.2%
20	Jordan	5	1.2%	55	Iceland	1	0.2%
21	Singapore	5	1.2%	56	Indonesia	1	0.2%
22	Belgium	4	1.0%	57	Iran	1	0.2%
23	Hong Kong	4	1.0%	58	Israel	1	0.2%
24	Mexico	4	1.0%	59	Jamaica	1	0.2%
25	Turkey	4	1.0%	60	Jersey	1	0.2%
26	Denmark	3	0.7%	61	Lebanon	1	0.2%
27	Malaysia	3	0.7%	62	Lithuania	1	0.2%
28	New Zealand	3	0.7%	63	Malta	1	0.2%
29	Niger	3	0.7%	64	Oman	1	0.2%
30	Nigeria	3	0.7%	65	Peru	1	0.2%
31	Pakistan	3	0.7%	66	Puerto Rico	1	0.2%
32	Poland	3	0.7%	67	Qatar	1	0.2%
33	Portugal	3	0.7%	68	Serbia	1	0.2%
34	Thailand	3	0.7%	69	Uruguay	1	0.2%
35	Austria	2	0.5%				

**Table 4:** Ten research topics classified by LDA.

Topic (Ranked by the Highest Volume in the Last 5 Years)	Research Topics	*n* (%)
middleic 1	Asthma control and education interventions for asthmatics	355 (21.8)
middleic 2	Development of medication for persistent asthma and allergic asthma	238 (14.6)
middleic 3	Association between diet and exercises and quality of life of people with asthma	167 (10.3)
middleic 4	Validation of questionnaires and evaluation of instruments	308 (18.9)
middleic 5	Impact of psychological and environmental factors on asthma-related quality of life	232 (14.3)
middleic 6	Asthma care and intervention for children and adolescents	160 (9.8)
middleic 7	Drugs for refractory asthma, eosinophilic asthma and non-eosinophilic asthma	71 (4.4)
middleic 8	Quality of life of asthmatics’ caregiversAsthma-related quality of life among the elderly	36 (2.2)
middleic 9	Relationship between allergens, allergic rhinitis and asthma status	52 (3.2)
middleic 10	Impact of non-medical treatment on health outcomes	7 (0.5)

LDA: Latent Dirichlet Allocation.
